# Simulation training approaches in intracranial aneurysm surgery—a systematic review

**DOI:** 10.1007/s10143-023-01995-5

**Published:** 2023-05-03

**Authors:** Fredrick J. Joseph, Hanne E. R. Vanluchene, David Bervini

**Affiliations:** 1 Image Guided Therapy, ARTORG Center for Biomedical Engineering Research, University of Bern, Bern, Switzerland; 2 Department of Neurosurgery, Bern University Hospital and University of Bern, Bern, Switzerland

**Keywords:** Clipping, Intracranial aneurysm, Medical education, Neurosurgery, Training

## Abstract

**Background:**

With the increasing complexity and decreasing exposure to intracranial aneurysm surgery, training and maintenance of the surgical skills have become challenging. This review elaborated on simulation training for intracranial aneurysm clipping.

**Methods:**

A systematic review was performed according to the PRISMA guidelines to identify studies on aneurysm clipping training using models and simulators. The primary outcome was the identification of the predominant modes of the simulation process, models, and training methods associated with a microsurgical learning curve. The secondary outcomes included assessments of the validation of such simulators and the learning capability from the use of such simulators.

**Results:**

Of the 2068 articles screened, 26 studies met the inclusion criteria. The chosen reports used a wide range of simulation approaches including ex vivo methods (*n* = 6); virtual reality (VR) platforms (*n* = 11); and static (*n* = 6) and dynamic (*n* = 3) 3D-printed aneurysm models (*n* = 6). The ex vivo training methods have limited availability, VR simulators lack haptics and tactility, while 3D static models lack important microanatomical components and the simulation of blood flow. 3D dynamic models including pulsatile flow are reusable and cost-effective but miss microanatomical components.

**Conclusions:**

The existing training methods are heterogenous and do not realistically simulate the complete microsurgical workflow. The current simulations lack certain anatomical features and crucial surgical steps. Future research should focus on developing and validating a reusable, cost-effective training platform. No systematic validation method exists for the different training models, so there is a need to build homogenous assessment tools and validate the role of simulation in education and patient safety.

## Introduction

In recent decades, the use of noninvasive cerebral vascular imaging has increased, resulting in greater detection of cerebral aneurysms [1, 2]. Nowadays, these aneurysms are increasingly treated endovascularly [2-4], even if certain complex cases are better suited for microsurgical clipping. The number of surgical clipping procedures is reducing [2, 5], providing fewer opportunities for neurosurgery trainees to practice clipping. This should be compensated so younger trainees are able to train for aneurysm clipping cases that are unsuited for endovascular treatment.

Although simulated training methods for minimally invasive procedures exist and are available in other specialties like orthopedics [6] and abdominal interventions [7], the simulation of microsurgical aneurysm clipping has only been thoroughly investigated in the last decade. It has been shown that excellent neurovascular training can still be obtained with a dedicated residency program and by improving skills using training models [5]. The use of 3D training models improves the understanding of relevant surgical anatomy as they provide knowledge of spatial relationships and a sense of depth [2]. Such models help residents to develop basic skills in aneurysm clipping [3, 8]. With these training models, residents can start practicing clipping procedures at an early stage of their residencies [9]. Using simulation models reduces the chance of errors during surgery and refines the surgical strategy, which benefits patient safety [3, 8].

This study reviews different state-of-the-art approaches for training simulations and compares them with benchtop models, thereby checking if they improve preoperative assistance and medical knowledge among residents. We focused on studies regarding intracranial aneurysm clipping simulation for training neurosurgeons in educational or clinical settings. The aim was to determine the effectiveness and usability of different training methods, as well as the possibility of obtaining hands-on experience. The reported strengths and limitations of each training method were determined along with the fidelity and feasibility of extensive training with the existing methods. The quality of training was also investigated to determine if current training methods can sufficiently prepare practitioners to treat cerebral aneurysms.

## Materials and methods

A systematic review was performed to identify research works on intracranial aneurysm clipping training using models and patient simulators. The search adhered to the PRISMA guidelines for reporting systematic reviews [10].

### Search strategy

The following electronic databases were searched from their inception until 21 September 2022: MEDLINE, Embase, PubMed, Cochrane, and PsycInfo. Each database search was conducted based on information source logic using Medical Subject Headings (MeSH/EMTREE) and Text Words. The search strategy used for MEDLINE is represented in Table 1; a similar search strategy was used for the other databases.

**Table 1:** Search strategy (MEDLINE)

References	Search term
1	Intracranial aneurysm
2	exp Neurosurgery/ or exp Internship and Residency/
3	exp Learning/ or exp Learning Curve/
4	exp Simulation Training / or exp Education, Medical/
5	exp Models, Anatomic/ or exp Printing, Three-Dimensional/
6	exp Virtual Reality/ or exp Computer Simulation/ or exp User-Computer Interface/ or exp Computer-Assisted Instruction/
7	exp Imaging, Three-Dimensional/ or exp Phantoms, Imaging/
8	exp Haptic Technology/
9	exp Clinical Decision-Making/ or exp Decision Making/ or exp Decision Support Systems, Clinical/ or exp Decision Support Techniques
10	exp Endovascular Procedures/ or exp Stents/ or exp Embolization, Therapeutic/
11	2 or 3 or 4 or 5 or 6 or 7 or 8 or 9
12	1 and 11
13	12 not 10

### Inclusion and exclusion criteria

Studies on the development and evaluation of simulation setups for aneurysm clipping training and those that verified the training abilities were considered. Studies based on computer simulations of fluid dynamics and endovascular procedures for aneurysm treatment were excluded. When multiple papers by the same authors and research groups covering the same topic with a similar scope were found, the most recent article was included. To evaluate the true effectiveness of the training approaches, only studies that involved neurosurgeons and/or residents as participants using the model and measured their training outcomes were included. Articles were not excluded based on publication year or language. Only articles on simulations that contributed to a learning process, improved surgical skills, or furthered medical education were included.

### Study selection and data extraction

No document restrictions or methodology filters were applied to the primary search. Duplicate records were removed when EndNote 20 software (Clarivate Analytics) searches were combined. Comments, reports, technical notes, letters, and videos were also removed. Initially, two authors (FJ and HV) independently screened all studies for relevance and eligibility based on the title and abstract. The authors resolved disagreements through discussion and consensus. Articles remaining after filtering based on the abstract were considered for full-text evaluation. Two authors (FJ and HV) completed several independent rounds of verification before deciding which studies to consider for review. Finally, a secondary search was conducted by two authors (FJ and HV) by scanning the reference lists of the retrieved articles.

### Information synthesis

The included references were divided into different groups according to the simulation method of the aneurysm and neighboring anatomies as seen in Table 2.

**Table 2:** Different types of training approaches

Category	Definition
Ex vivo: cadavers	Training on pathology created using 3D printing or made using graft vessels found/injected into a cadaver head with/without blood-like flow
Ex vivo: human placenta	Training on an aneurysm/bulge structure created on human placenta with/without blood-like flow
Ex vivo: chicken specimen	Training on an aneurysm/bulge structure found/created on vessels from chicken/turkey limbs with/without blood flow
Virtual reality	Pathology and/or anatomy model built using a virtual reality environment with/without haptics, tactility, and blood flow emulation
3D static simulators	Pathological and/or anatomical model built using a 3D printer and/or additive manufacturing technology as a solid object without blood flow
3D dynamic simulators	Pathological and/or anatomical structures/models built using a 3D printer and/or additive manufacturing technology as solid and/or hollow realistic objects with pulsatile/dynamic blood flow as a tabletop/benchtop system

As no quantitative studies were found, the following aspects of each paper are descriptively highlighted:

1. *Summary of the study method:* This included the number and type of simulated intracranial aneurysms, the number and background of participants if mentioned, performed validity assessment, reported learning/simulation outcomes, and reported disadvantages. Regarding validity, it was noted whether the training method was tested for face validity, content validity, construct validity, feasibility, predictive validity, or concurrent validity. Face validity is the extent to which the simulation resembles real life, content validity refers to the extent to which the simulation is complete and accurate, and construct validity is the extent to which performance on the simulator discriminates between novices and experts [11]. Feasibility refers to the measurement of whether the simulation is possible, predictive validity involves checking how well the performance on a simulator predicts future performance, and concurrent validity is the extent to which performance on a simulator correlates with best practices.
2. *Extent of simulation training:* This included a detailed description of the simulation training method assessing the following points:
  - Inclusion of haptics, meaning that the sense of touch was incorporated into the model through the provision of force feedback [12]
  - Inclusion of tactility, which is a component of haptics that focuses on the sensation of pressure, leading to the identification of object features like holes and surface friction [12]
  - Presence of blood or dynamic flow in the pathology, and if this was pulsatile flow
  - Replication of true scale (one-to-one geometrical replication) of the anatomy and pathology without scaling the dimensions, and if this was controlled
  - Realism of the simulation, accomplished when more than 80% of the participants in the study group were convinced of the real-life perception
  - Simulation steps in relation to the clinical workflow (craniotomy, subarachnoid dissection, approach through subarachnoid spaces, vascular control, aneurysm exposure, clipping, post-clipping investigation, confirmatory inspection)
  - Access and availability of simulation type: for ex vivo simulations, the availability of tissues was checked, and for virtual reality (VR) and 3D simulators, the time of model production was a determining factor
  - Model reusability
  - Model cost
  - Country of study origin or author’s country, to reconsider access and availability
3. *Applicability of the simulation:* This included a summary of the demonstrated simulation modality for each reference and an evaluation of the possibility of preparing the simulation model in a shorter time for presurgical training. The demonstrated simulation modalities consisted of the following options:
  - Lecture and demonstration, meaning that a lecture was given or an experienced neurosurgeon demonstrated the clipping procedure on the model
  - Workshop/periodic hands-on-experience, wherein the participants had a special extended opportunity to test out the model
  - Residency/frequent training, meaning that the model was used for training more often
  - Preoperative planning, wherein the study shows demonstrated the use of the model as a presurgical planning or training tool

## Results

### Study characteristics

**Fig. 1:**
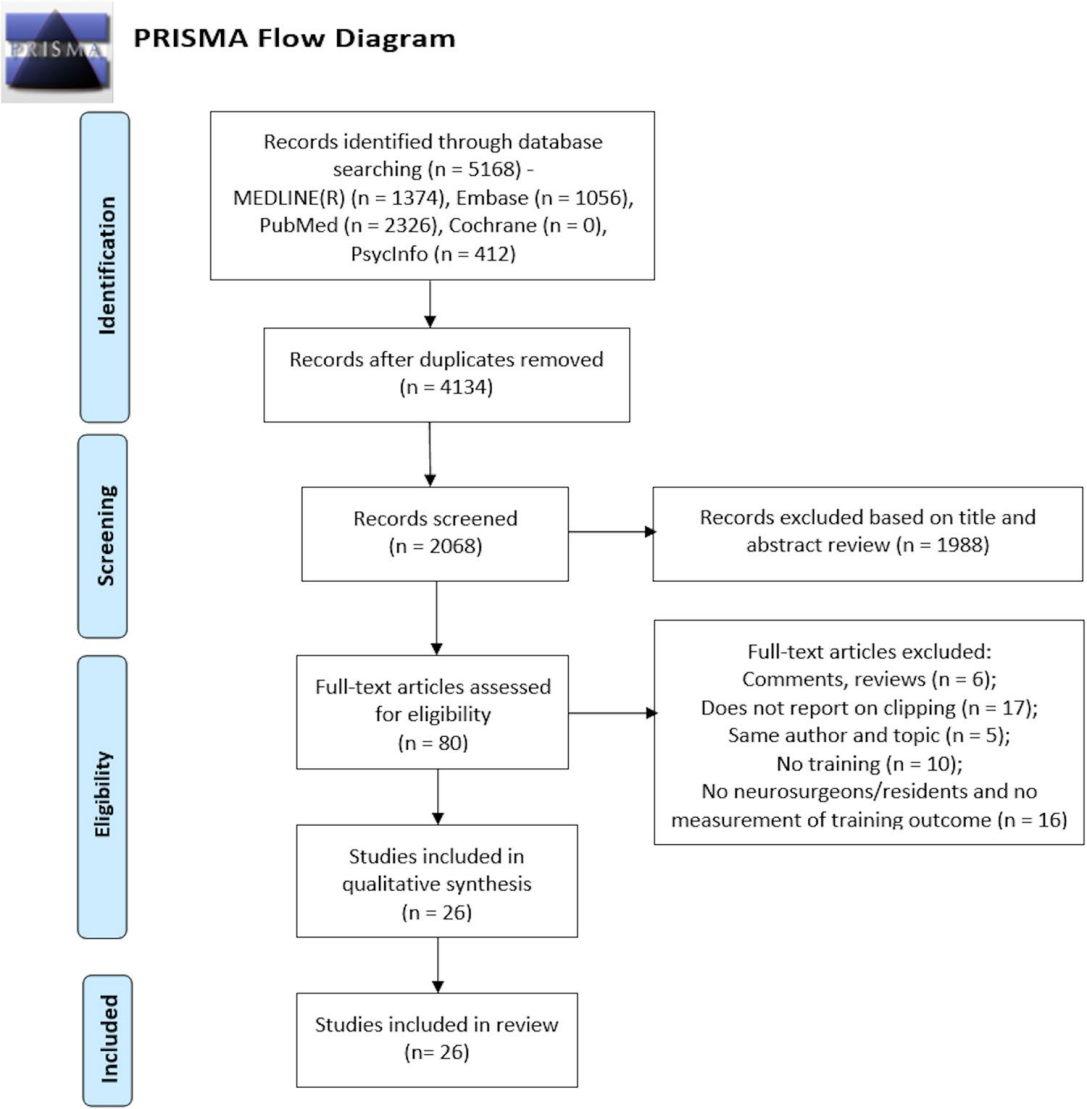
PRISMA flow chart of the selection process for the included studies

In total, 5168 references were retrieved from the databases, of which 1034 duplicates were removed. According to the search strategy represented in Fig. 1, 26 articles met the inclusion criteria. Two studies were added to the category “ex vivo: cadavers” [13, 14], one study was added to “ex vivo: human placenta” [15], and three studies were added to “ex vivo: chicken wings” [16-18]. “Virtual reality” contained 11 studies [19-29], “3D static simulators” had six [30-35], and “3D dynamic simulators” had three [9, 36, 37].

The simulation approach, study design, and validation method of each reference were assessed; the findings are summarized in Tables 3, 4, and 5.

**Table 3:** Summary of the study methods

Study	Type and number of intracranial aneurysms simulated	Number of study participants and background			Validity assessments performed	Learning/simulation outcomes reported	Disadvantage(s) reported
		NS	NR	GR			
Ex vivo*: cadavers*							
de Oliveira et al. 2018 [13]	Various (*n* = 30, in placenta); MCA (*n* = 4, in cadavers)	12	9	-	Face validity (descriptive questionnaire); Content validity (surgical videos of residents with different training, assessed by a descriptive questionnaire, blinded) used to assess predictive and concurrent validity	Residents with the placenta model performed better; the placenta model required more extended time to complete the task; the placenta model was more effective in simulating sylvian fissure splitting, bipolar coagulation of oozing micro vessels, and aneurysm neck and dome dissection; the cadaver model was superior for the simulation of intraoperative rupture and reproduction of real anatomy	Limited availability of fresh cadavers; Need for an adequate perfusion machine
Aboud et al. 2015 [14]	Various (*n* = 59)	89	203	-	Face validity and feasibility (descriptive questionnaire)	Participants agreed that the model was a valid simulation of live surgery conditions and a realistic simulation of clipping and intraoperative rupture; the model was appropriate for practicing teamwork during intraoperative crisis	Limited availability of cadavers; poor quality of tissues and properties of artificial blood
Ex vivo*: human placenta*							
Belykh et al. 2017 [15]	NM	10	10	10	Face and content validity (Aneurysm Clipping Participant Survey); Construct validity (OSAACS tool)	Face validity (models were rated as reasonable replications of real surgery); content validity (models showed improvements in microdissection technique, use of clips, and surgical technique when applied to patients); construct validity (mean OSAACS increased with experience)	Bias (raters not blinded); subjective face and content validity; Absence of control group; limited time to use placenta; no simulation of skull base approaches, clinoid process removal, and surgical vascular anatomy
Ex vivo*: chicken*							
Carlos et al. 2022 [16]	NM	10	-	-	Face and content validity (descriptive questionnaire); Construct validity (Doppler probe and visual inspection)	Neurosurgeons agreed that the simulator and the trained skills were comparable to clipping and believed the simulator could improve patient safety	Construct validity not assessed; Clinical effectiveness not determined
Belykh et al. 2021 [17]	Various (*n* = 3)	8	8	-	Face and content validity (Aneurysm Clipping Participant Survey); Construct validity (OSAACS tool)	Face validity (the model was scored as replicating actual aneurysm clipping and its difficulty in a manner comparable to real surgery); content validity (the model was rated as improving clip-applier-handling skills when working with patients); construct validity (experienced neurosurgeons performed significantly better than trainees)	No microdissection modeled; Limited production of models; No cranial nerves or venous anatomies modeled; Pulsatile perfusion simulated manually
Giovani et al. 2019 [18]	Various (*n* = 296)	NM	NM	NM	Content validity (satisfaction questionnaire)	Aneurysm models were successfully developed; neurosurgeons rated the models as useful	No simulation of brain anatomy; No simulation of arachnoid dissection
*Virtual reality*							
Perin et al. 2021 [19]	Various (*n* = 11)	4	3	-	Face and content validity (descriptive questionnaire); Construct validity (ad hoc clipping score, modified Rankin Scale for control and VR group)	No differences were found in surgical outcomes, complications, and duration between two groups; a high degree of appreciation was shown by participants concerning simulator rehearsal; junior neurosurgeons improved their performance with the simulator after a debriefing session	No aneurysm manipulation with haptic feedback and deformation feature; No simulation of aneurysm dissection and neck exposure; No manipulation of brain parenchyma; Small study sample; Expensive; Limited diffusion
Steineke & Barbery 2021 [20]	Various (*n* = 10)	NM	NM	NM	Construct validity (comparison of different factors between the control and VR group)	Mean procedure time was significantly lower for the VR group; no significant difference was observed in the Charlson Comorbidity Index score between groups	Lack of validated complexity scoring; No comparison of clip correction and occlusion rates
Teodoro-Vite et al. 2021 [21]	Various (*n* = 2)	6	-	6	Face and content validity (descriptive questionnaire)	Neurosurgeons performed more gestures of brain tissue retrieval; residents exerted higher maximum force; Neurosurgeons scored usability and realism as high	Low number of participants; Spatial registration manually performed; Parenchymal tissue retracted more than in reality; Instrument mechanisms needed adaptation to haptic devices
Gmeiner et al. 2018 [22]	MCA (*n* = 4)	14	4	-	Face and content validity (descriptive questionnaire); Predictive validity (comparison of surgical and virtual clipping)	The simulator improved neurosurgeons’ anatomic understanding; participants rated the simulation of head positioning and craniotomy as realistic; participants agreed to integrate the simulator into neurosurgical education	No arachnoid dissection simulated; only MCA aneurysm simulated
Shono et al. 2018 [23]	Various (n = 8)	3	-	-	Face and content validity (questionnaire); Predictive validity (comparison of surgical and virtual clipping)	A clipping simulator was successfully developed; the simulation findings coincided highly with intraoperative findings	Quality of model linked to quality of medical images; high workload to build the system
Chugh et al. 2017 [24]	Various (*n* = 25)	2	2	-	Content and construct validity (comparison of surgeries with and without virtual planning, blinded)	The mean values of the number of clip attempts, total operative time, ratio of clip attempts to clips used, and time per clip attempt were lower in the VR group (only time per clip was statistically significant)	Bias (varying case complexity between groups); small sample size
Kockro et al. 2016 [25]	Various (*n* = 115)	9	-	-	Predictive validity (comparison of surgical and virtual clipping, modified Rankin scale)	Spatial understanding, clip preselection, and positioning improved; Less experienced neurosurgeons also had good outcomes	Selection bias
Alaraj et al. 2015 [26]	MCA (*n* = 1)	-	17	-	Face and content validity (descriptive questionnaire)	Participants found the simulation useful for preparing for real-life surgery; 2/3 participants found that the anatomic details closely resembled real operative anatomy; participants found the simulation useful for preoperative surgical rehearsal and training; 1/3 found the haptic feedback realistic	Validity determined by residents through a questionnaire
Marinho et al. 2014 [27]	Various (*n* = 10)	2	-	-	Feasibility (subjective assessment); Predictive validity (comparison of surgical and virtual clipping)	The visual similarity of the virtual scene and the operative view was excellent; identification of vascular structure was accurate in 90% of cases; neurosurgeons rated the simulation helpful for planning; The simulation findings coincided highly with intraoperative findings	High risk of analysis bias; manipulation of clip appliers not intuitive; Imaging artifacts limited the simulation; no tactile and haptic feedback; no training advantage
Bambakidis et al. 2013 [28]	MCA (*n* = 1)	NM	NM	NM	Content and construct validity (videos to compare surgery with and without virtual planning, blinded)	Microsurgical time and clipping attempts reduced	NA
Wang S. et al. 2012 [29]	Various (*n* = 57)	NM	NM	NM	Predictive validity (comparison of surgical and virtual clipping)	VR led to a more concise operative procedure and more confident surgeons; vessels were clearly visualized; estimation of bone removal at clinoid was possible	No separation or retraction of cranial nerves and blood vessels; no true simulation of clipping; lack of haptic feedback; no difference in feeling between tissues
*3D static simulators*							
Mery et al. 2021 [30]	ACOM (*n* = 1); BA (*n* = 1)	-	32	-	Face and content validity (descriptive questionnaire); Construct validity (comparison of the answers of different groups)	Face validity (participants highly agreed on the regional anatomy and arrangement of the simulator and instruments to represent surgical reality); content validity (participants agreed that the model allowed proper visualization and manipulation under microscopic vision and good performance of craniotomy)	Absence of soft tissues; absence of flow-based vascular anatomy
Wang L. et al. 2018 [31]	MCA (*n* = 8)	-	6	-	Face and content validity and feasibility (descriptive questionnaire)	Clips chosen in the simulation were similar to clips used during actual surgery; participants agreed that the model improved residents’ understanding of the relationship between the aneurysm and parent artery and that the simulator was helpful in training	Brain not included; no presence of intra-arterial thrombus; aneurysm wall thickness and calcified or blister variants not included
Wang J.L. et al. 2018 [32]	Various (*n* = 13)	-	7	15	Face and content validity (descriptive questionnaire); Content validity (patient follow-up with modified Rankin scale)	A model matching medical images was successfully developed; participants reported that the model helped in understanding aneurysm anatomy and improving surgical skills and was clinically applicable, and that the training course was useful	No optic chiasm, cranial nerves, skulls, or brains included
Mashiko et al. 2017 [33]	MCA (*n* = 3)	2	-	4	Content validity (descriptive questionnaire); Construct validity (descriptive questionnaire for senior neurosurgeons to assess residents’ skills before and after the training course)	Trainees succeeded in performing the simulation in line with actual surgery; trainees’ skills improved upon completion of the training with the model	Imperfect feel of materials; inconvenient steps to fabricate an arachnoid; no real tactile feedback; Subjective assessment
Ryan et al. 2016 [34]	Various (*n* = 9)	-	14	-	Face and content validity (descriptive questionnaire)	A patient-derived simulacrum was developed; participant reports suggested the potential to enhance current educational programs and showed model efficacy	Cranial nerves missing; no arachnoid dissection simulated; overaccentuated wall thickness in hollow vessel portions
Kimura et al. 2009 [35]	Various (*n* = 11)	NM	NM	NM	Predictive validity (comparison of surgical and simulated clip selection)	Clips during surgery and the simulation were applied in the same direction and configuration; the model led to the improved handling of instruments and understanding of configuration and neck occlusion	Surrounding tissues around aneurysm not included; Long preparation time
*3D dynamic simulators*							
Joseph et al. 2020 [36]	MCA (*n* = 1)	9	16	-	Face and content validity (descriptive questionnaire); Construct validity (simulation videos compared between participants by a blinded expert neurosurgeon)	The simulator was reliable and useful for training; participants reported that it was a superior alternative to conventional neurosurgical training methods	Skin, galea, meninges, veins, and arachnoid missing; No complete surgical workflow could be reproduced
Leal et al. 2019 [37]	MCA (*n* = 1)	4	-	-	Predictive validity (comparison of surgical and simulated clip selection)	The clip during simulation and surgery was the same; the model was helpful for training and planning	No simulation of delicate maneuvers like brain dissection and retraction
Liu et al. 2017 [9]	Various (*n* = 4)	6	-	4	Face and content validity (descriptive questionnaire)	An anatomically personalized cerebral aneurysm simulator was successfully developed; residents developed a better understanding of intracranial aneurysms and gained experience in coping with vessels during surgery; participants agreed on the value of training with the model	NA

*ACA*, anterior cerebral artery aneurysm; *ACOM*, anterior communicating artery aneurysm; *BA*, basilar artery aneurysm; *GR*, general resident; *IC-PC*, internal carotid-posterior communicating artery aneurysm; *MCA*, middle cerebral artery aneurysm; *NA*, not assessed; *NM*, not mentioned; *NR*, neurosurgical resident; *NS*, neurosurgeon; *OSAACS*, Objective Structured Assessment of Aneurysm Clipping Skills; *PCOM*, posterior communicating artery aneurysm; *VA*, vertebral artery aneurysm. If various types of intracranial aneurysms were simulated, at least two of the following kinds of aneurysms were included: MCA, ACOM, ACA, PCOM, IC-PC, and VA.

**Table 4:** Extent of simulation training

Study	Characteristics of simulation training method							Access to and availability of simulation type	Model reusability	Cost	Country
	Haptics	Tactility	Presence of blood or dynamic fluid flow into the pathology	Pulsatile flow	Replication of true-scale anatomy and pathology	Real-life simulation according to participants (> 80%)	Simulation steps included				
Ex vivo*: cadavers*											
de Oliveira et al. 2018 [13]	P	P	P	P	NM	NA	Sylvian fissure dissection, aneurysm approach, aneurysm clipping, rupture	Very low availability of cadavers	N	NM	Brazil
Aboud et al. 2015 [14]	P	P	P	P	NM	Y	Sylvian fissure dissection, aneurysm approach, aneurysm clipping, rupture	Very low availability of cadavers	N	> $1000	USA, Egypt, Taiwan, Germany
Ex vivo*: human placenta*											
Belykh et al. 2017 [15]	P	P	P	P	NM	N	Sylvian fissure dissection, aneurysm approach, aneurysm clipping	Informed consent from obstetric patients needed	N	NM	USA, Russia
Ex vivo*: chicken*											
Carlos et al. 2022 [16]	P	P	P	P	NM	N	Sylvian fissure dissection, aneurysm approach, aneurysm clipping	Bovine and chicken samples easily accessible	N	$19.58	Argentina
Belykh et al. 2021 [17]	NM	NM	P	P	NM	Y	Aneurysm clipping	Turkey arteries readily available	N	$10	USA
Giovani et al. 2019 [18]	P	P	A	A	Y	NA	Aneurysm clipping	Chicken wings easily available	N	NM	Romania, India
*Virtual reality*											
Perin et al. 2021 [19]	A	A	A	A	NM	NA	Craniotomy, aneurysm approach, aneurysm clipping	Segmentation process < 1 h	Y	$250,000	Italy
Steineke & Barbery 2021 [20]	NM	NM	A	A	NM	NA	Craniotomy, aneurysm clipping	5–15 min to render each case	NA	NM	USA
Teodoro-Vite et al. 2021 [21]	P	P	A	A	Y (but not proven)	N	Sylvian fissure dissection, aneurysm approach, aneurysm clipping	NM	NA	NM	Mexico
Gmeiner et al. 2018 [22]	P	P	P	A	NM	N	Craniotomy, aneurysm clipping	1–2 h to transfer CTA (computed tomography angiography) or 3D angiography into simulator	Y	NM	Austria
Shono et al. 2018 [23]	A	A	A	A	NM	NA	Sylvian fissure dissection, aneurysm approach, aneurysm clipping	A maximum of 5 h to build the simulator for a cerebral aneurysm clipping case and a maximum of 7 h to create model fusion	Y	NM	Japan
Chugh et al. 2017 [24]	P	NM	A	A	NM	NA	Aneurysm clipping	NM	Y	NM	USA
Kockro et al. 2016 [25]	NM	NM	A	A	NM	NA	Craniotomy, aneurysm clipping	NM	Y	NM	Switzerland, Germany
Alaraj et al. 2015 [26]	P	P	P	A	NM	N	Craniotomy, aneurysm approach, aneurysm clipping, rupture	NM	Y	NM	USA
Marinho et al. 2014 [27]	A	A	A	A	NM	NA	Aneurysm clipping	NM	NA	NM	France
Bambakidis et al. 2013 [28]	A	A	A	A	NM	NA	Aneurysm clipping	NM	NA	$250,000	USA
Wang S. et al. 2012 [29]	A	A	A	A	NM	NA	Aneurysm clipping	NM	NA	NM	China
*3D static simulators*											
Mery et al. 2021 [30]	NM	NM	A	A	NM	Y	Craniotomy, aneurysm clipping	NM	N	$2500 (for installation), $180 (for further training sessions)	Chile, USA
Wang L. et al. 2018 [31]	NM	NM	A	A	Y	N	Craniotomy, aneurysm clipping	A maximum of 20 h of process time	NA	$350	China
Wang J.L. et al. 2018 [32]	NM	NM	A	A	Y	NA	Aneurysm clipping	NM	Y	$22	China
Mashiko et al. 2017 [33]	A	A	A	A	NM	NA	Aneurysm approach, aneurysm clipping	3 days	Y	$200	Japan
Ryan et al. 2016 [34]	NM	P	A	A	Y (but not proven)	Y	Aneurysm approach, aneurysm clipping	NM	Y	< $1000	USA
Kimura et al. 2009 [35]	NM	NM	A	A	NM	NA	Aneurysm clipping	3–7 days	Y	$400	Japan
*3D dynamic simulators*											
Joseph et al. 2020 [36]	P	P	P	P	Y (but not proven)	Y	Aneurysm approach, aneurysm clipping	NM	Y	NM	Switzerland
Leal et al. 2019 [37]	NM	NM	P	A	N	NA	Aneurysm clipping	Production time of 4–5 h	NA	$100	Brazil
Liu et al. 2017 [9]	P	A	P	P	NM	NA	Aneurysm approach, aneurysm clipping	8–55 min to fabricate vessel depending on complexity	NA	$25	China

*A*, absent; *N*, no; *NA*, not assessed; *NM*, not mentioned; *P*, present; *Y*, yes

**Table 5:** Applicability of simulation

Study	Demonstrated applicability of specified simulation modality				Preparation of simulation model in a shorter time for pre-surgical training
	Lecture and demonstration	Workshop/ one-time hands-on experience/ periodical hands-on experience	Residency program/training	Preoperative planning	
Ex vivo*: cadavers*					
de Oliveira et al. 2018 [13]	NA	D	D	NA	NA
Aboud et al. 2015 [14]	D	D	D	NA	NA
Ex vivo*: human placenta*					
Belykh et al. 2017 [15]	NA	D	NA	NA	NA
Ex vivo*: chicken*					
Carlos et al. 2022 [16]	NA	D	NA	NA	NA
Belykh et al. 2021 [17]	NA	D	NA	NA	NA
Giovani et al. 2019 [18]	NA	D	NA	NA	NA
*Virtual reality*					
Perin et al. 2021 [19]	D	D	NA	D	NA
Steineke & Barbery 2021 [20]	NA	NA	NA	D	D
Teodoro-Vite et al. 2021 [21]	NA	D	NA	NA	NA
Gmeiner et al. 2018 [22]	D	D	NA	NA	NA
Shono et al. 2018 [23]	NA	NA	NA	D	NA
Chugh et al. 2017 [24]	NA	NA	NA	D	NA
Kockro et al. 2016 [25]	NA	NA	NA	D	D
Alaraj et al. 2015 [26]	NA	D	NA	NA	NA
Marinho et al. 2014 [27]	NA	NA	NA	D	NA
Bambakidis et al. 2013 [28]	NA	D	NA	NA	NA
Wang S. et al. 2012 [29]	NA	D	NA	D	NA
*3D static simulators*					
Mery et al. 2021 [30]	NA	D	NA	NA	D
Wang L. et al. 2018 [31]	NA	D	NA	D	NA
Wang J.L. et al. 2018 [32]	D	D	NA	NA	D
Mashiko et al. 2017 [33]	D	D	NA	NA	NA
Ryan et al. 2016 [34]	D	D	NA	NA	D
Kimura et al. 2009 [35]	NA	D	D	D	NA
*3D dynamic simulators*					
Joseph et al. 2020 [36]	NA	D	NA	NA	NA
Leal et al. 2019 [37]	NA	D	NA	NA	NA
Liu et al. 2017 [9]	NA	D	NA	NA	NA

*D*, demonstrated; *NA*, not assessed

#### Ex vivo* specimens*

Artificial aneurysms were created based on human placental vasculature, chicken wing or thigh vessels, or venous grafts from cadavers’ necks. Carlos et al. (2022) [16] introduced artificial aneurysms into bovine brains, while Aboud et al. (2015) [14] introduced them into 23 living cadavers between 2009 and 2014.

#### VR

Different setups were used for the VR simulations. Some studies used a surgical rehearsal platform such as Surgical Theater (Cleveland, OH, USA) [19, 20, 24, 28] or the patient-specific environment Dextroscope (Bracco Diagnostics, Monroe Township, NJ, USA) [25, 29]. Marinho et al. (2014) [27] created a virtual operative environment with the open-source software Blender. Alaraj et al. (2015) [26] developed a VR simulator using the ImmersiveTouch (Chicago, IL, USA) platform. Shono et al. (2018) [23] made use of the integrated development environment of unity to create the simulation. Several studies created a custom-designed VR setup [21, 22].

#### 3D static simulators

Pathology is replicated using 3D printers. Apart from the aneurysm and the neighboring vessels, some studies also included the skull and/or brain. The studies focused on creating hollow aneurysms [31] or a total hollow vasculature [32-34] in the model by using elastic 3D-printed material [30, 35].

#### 3D dynamic simulators

All dynamic simulators replicated the aneurysm and vasculature with hollow elastic vessels, allowing for blood flow simulation. Leal et al. (2019) [37] injected a physiological solution into the aneurysm to represent blood flow. Joseph et al. (2020) [36] used a custom-designed pulsatile pump, and Liu et al. (2017) [9] used a blood flow driver to simulate pulsatile blood flow.

### Method of assessment

The assessment method for the simulation approach varied across articles. Most articles included a subjective, descriptive questionnaire to assess the face validity, content validity, and/or feasibility [9, 13-19, 21-23, 26, 30-34, 36]. Some articles on VR simulation compared a control group with a VR group; the control group used only conventional presurgical planning tools, while the VR group used a VR simulator to perform presurgical planning [19, 20, 24, 28]. A comparison of aneurysm complexity, surgical time, and surgical outcomes was made [19, 20], or surgical videos were recorded, which allowed for the assessment of the clipping itself by an experienced neurosurgeon [24, 28]. In other studies that conducted a review, a senior neurosurgeon assessed clipping performance based on neurosurgical videos [13] or microscope videos of the simulation [36] or by watching the residents’ performance [33]. Belykh et al. (2017) [15, 17] used an objective tool to assess the construct validity, namely the Objective Structured Assessment of Aneurysm Clipping Skills (OSAACS) tool, which measures specific operative nuances of aneurysm clipping surgery for which an assessor determines the performance of residents. Another assessment method used by some references involved a comparison of the virtual clipping and the surgical clipping based on the clip used, clip position, and the number of clips [22, 23, 25, 27, 29, 35, 37].

### Quality assessment

The articles varied in quality, which was reflected in their assessment methods. Some references only included a descriptive questionnaire for assessment, which is a subjective assessment leading to lower quality outcomes. Most references did not mention potential bias in their study. Belykh et al. (2017) [15] noted the presence of some bias as the raters were not blinded to the participants. Kockro et al. (2016) [25] reported selection bias as the complex aneurysm cases were mostly handled by experienced neurosurgeons. Marinho et al. (2014) [27] reported analysis bias due to the subjectivity of the limited number of participants. Some studies used blinding to decrease the risk of bias. In the studies by Oliveira et al. (2018) [13] and Chugh et al. (2017) [24], the reviewer was blinded as to whether conventional planning or simulation was used before the surgery. In the study by Joseph et al. (2020) [36], the microscope videos were anonymized so that the reviewer was blinded to the participant’s identity. The study by Bambakidis et al. (2013) [28] was double blinded. Additional sources of bias are observed by the authors. Some studies included only a limited number of participants [23, 24, 27, 31, 33, 37], leading to bias regarding the level of experience. Other studies simulated only a low number of aneurysms [21, 26, 28, 30, 36, 37], resulting in a bias toward the complexity of the aneurysm case. It is not sure if the studies would have the same outcomes with surgeons of different experience and with other aneurysm complexity cases.

## Discussion

### Summary

The creation of good training methods for the clipping of cerebral aneurysms is crucial for the proper education of trainees. Different methods have been investigated to recreate aneurysms based on ex vivo specimens, including the use of chicken wings, pigs, cadaveric brains, 3D-printed aneurysms implanted in cadavers, and cadaveric cow craniums [38-42]. Aboud et al. (2015) [14] and Oliveira et al. (2018) [13] reported using human cadavers with and without blood flow and dynamic behavior. These methods provided excellent haptic feedback and allowed the residents to train in multiple surgical steps, but the studies did not report definitive outcomes. Some cadavers with pathology were collected over an extended period and were difficult to perfuse. This type of setup is limited because cadavers are resource intensive and require special refrigeration, making them unsuitable for extended resident training programs. In addition, the studies did not report on the repeatability of procedures or the feasibility of patient-specific learning outcomes. The studies by Belykh et al. and others that used human placentas and chicken wings [15-18] included dissection and perfusion to replicate dynamic behavior but failed to replicate the surgical workflow during and after the clipping procedure. They included a structured performance assessment by an expert, which proved that the trainees had learned. Oliveira et al. (2018) [13] showed that residents training on placenta models performed better than residents training on cadavers or only having surgical videos available. However, without human-specific anatomy and pathological geometry in ex vivo models, these studies could not compare trainee performance to the clipping approach. In addition, preparing such ex vivo models is time-consuming [14], so they are unsuitable for presurgical planning and training.

VR systems do not have preservation problems, and they have been highly investigated for planning [43-45], focusing on force feedback, distance visualization, or a craniotomy approach [46-48]. Participants in VR studies could clip aneurysms multiple times, but training for the surgical experience was unrealistic although the simulation created an immersive environment. These simulators lack realistic haptic feedback, tactility, pulsatile flow, and real-life simulation [19, 23, 27-29]. Some VR studies implemented haptic feedback through haptic controllers for both hands, but participants scored haptic reality low [21, 22, 24, 26]. All VR studies could simulate aneurysm clipping, but none could include all other steps involved in a complete surgical workflow (Table 4). Availability, cost, and access to VR simulators created barriers to their use [19, 23]. The VR studies that compared a control group showed decreased surgical time when training with a VR simulator [20, 24, 28].

More practitioners are using 3D-printed models to understand clipping procedures in their education and training because technology has progressed rapidly and patient-specific approaches are made possible by producing models from patient images [49-54]. Hollow aneurysm models have been created, which allow for the performance of clipping [55, 56], but they lacked realistic haptic touch. The models did not introduce blood flow or surgical complexities. Because of these missing components, only a limited part of the surgical workflow could be simulated. Although static training models have these major limitations, an advantage is that 3D models can be reused for multiple training sessions if no craniotomy is performed.

The absence of the dynamic behavior of blood flow is a big disadvantage as it does not allow for the confirmation of a successful clipping. The clip should occlude the aneurysm completely at neck level, and parent arteries should not be compromised by the clip [57]. This can be confirmed when blood flow is available in the training model. Adding a contrast marker in the blood can facilitate the validation of correct clipping under a microscope. By using pulsatile flow, touching the dome and arteries can already give an idea of whether the clipping was successful, and the use of Doppler angiography can provide further confirmation.

This leads to dynamic 3D simulations being more realistic as they allow simulation of the blood flow [58, 59]. Joseph et al. (2020) [36] and Liu et al. (2017) [9] added pulsatile blood flow and realistic haptic feedback to their simulation models, allowing for a realistic simulation of the aneurysm approach and clipping as was reported by the participants of these studies. These dynamic models can be reused for multiple training sessions, making them a cost-effective option. Besides these advantages, the dynamic models are not realistic for the whole neurosurgical workflow as they also lack skin, meninges, and important microanatomical features such as cranial nerves, smaller arteries, and veins. Aneurysm clipping surgery is not only about the clipping itself but also involves the planning approach, strategy, and direction to safely reach the pathology without sacrificing major microanatomical structures. Therefore, the dynamic simulators are not sufficient to simulate important microsurgical steps. Future research should focus on simulations that include all anatomical and microanatomical structures to have a more valid representation of the complete surgery. Different technological methods could be combined to address some limitations, for example, creating a mixed environment using augmented reality technology and incorporating dynamic flow on VR system.

### Limitations

The heterogeneity of the reviewed studies made it difficult to perform direct comparisons. This is related to the variance in training and assessment methodology worldwide. A standard methodology scale could be used to objectively determine clipping scores. An effort for this was already performed by creating the Objective Structured Assessment of Aneurysm Clipping Skills (OSAACS) [15] and Skills Assessment in Microsurgery for Brain Aneurysms (SAMBA) [60] scoring systems. However, both still have limitations in assessing the complete aspects of aneurysm surgery. SAMBA focuses only on the aneurysm clipping aspects, while OSAACS focuses more on other intra-surgical steps and less on the clipping itself. Therefore, a combination of these two scoring systems should be developed. For future research, a consensus on assessment methods should be reached to compare the different studies. Implementing hands-on training is limited by different factors; the availability of the simulators, the supply chain, the replacement of head micro-anatomy when ruptured, the training site’s economic situation, and mentorship availability all play an important role in establishing the training environment [61-63]. No clear validation method is available for the training methods; therefore it is impractical for studies to prove their effectiveness and to make a valid comparison with other training methods. In the future, similar studies should focus on comparing different simulation methods and comparing them to a control group without simulation to be able to show effectiveness.

### Future directions

The overall significance of this systematic review lies in developing the latest knowledge on patient-specific realistic training approaches to be able to educate neurosurgeons and residents and help researchers and medical device manufacturers in designing, developing, and implementing simple and innovative systems in treatment practices. The key perspectives represented in this review could help stakeholders like surgical staff, hospital administration, medical universities, and training institutes to understand the evolution of training and the need to adapt to future directions during the development of policies and guidelines for the aneurysm training and treatment process.

## Conclusion

Numerous efforts have been made to create training simulations for cerebral aneurysm surgery. The existing training methods are very heterogenous and carry major potentials. However, they do not realistically simulate the complete microsurgical workflow for clipping cerebral aneurysms. The current simulations lack certain anatomical features and crucial surgical steps. Future research should focus on developing and validating a reusable, cost-effective, high-fidelity training platform. No systematic validation method exists for the different training models, so there is a need to build homogenous assessment tools and validate the role of simulation training in education, direct patient safety. and transferability to the clinics.
